# "The Hospital" Medical Book Supplement.—No. XX

**Published:** 1909-07-17

**Authors:** 


### "THE HOSPITAL" MEDICAL BOOK SUPPLEMENT.—No. XX.

CONTENTS.

**Tuberculosis—**
Rational Immunisation in the Treatment of Pulmonary Tuberculosis and Other Diseases ... 417
Tuberculin in Diagnosis and Treatment ... 417
**Diseases of the Heart—**
Graphic Methods in Heart Disease ... 418
**Tropical Medicine—**
Jamaica ... 418
**Nursing and Allied Subjects—**
Handbooks for Midwives and Maternity Nurses ... 419
Syllabus of Lectures on Home Nursing ... 419
**Eugenics—**
Parenthood and Race Culture ... 419
**Materia Medica and Therapeutics—**
Southall's Organic Materia Medica ... 419
How to Cut the Drug Bill ... 419
**Miscellaneous Items—**
The Interpretation of Radium... ... 420
The Frontiersman's Pocket-book ... 420
The Climate of Strathpeffer ... 420
Wastage of Child Life ... 420
Golden Rules of Anæsthesia ... 420

### TUBERCULOSIS.

RATIONAL IMMUNISATION IN THE TREATMENT OF PULMONARY TUBERCULOSIS AND OTHER DISEASES. By E. C. HORT, B.A., B.Sc., M.R.C.P. (London: John Bale, Sons and Danielsson, Ltd. 1909. Pp. 75. Price 3s. 6d. net.)

THE first main point upon which Dr. Hort insists is that we are all of us, nowadays, far too apt to think that the symptoms of any particular microbial disease are due to the bacteria or to their toxins. Dr. Hort would have us remember that the way in which many of the symptoms arise is by no means directly from the microbes or from the microbial toxins, but rather from abnormal or toxic substances produced by our own tissue cells as the result of the action of the bacteria upon the latter. In other words, in addition to any symptoms that may be due to the bacterial toxins, there are other symptoms due to our own abnormal tissue-toxins. Now modern vaccine treatment is directed almost entirely against the microbe or against the microbial toxin. No effort is being directly made to combat the toxins that are produced by the tissue cells that the microbes have attacked and damaged, and the opsonic method, quite apart from any intrinsic defects, is bound to be absolutely misleading. A rise in the opsonic index is at most an indication that the power of resisting the microbe in question is increasing; though even this conclusion is open to dispute. Of the many other points that Dr. Hort makes, we will mention only that which concerns evening-temperature charts. Dr. Latham has shown that the temperature charts in cases of tuberculosis afford most valuable evidence as to the good or bad progress of the case—evidence that can be relied upon more safely than can opsonic index counts. This is doubtless because opsonic counts afford evidence upon one point only—namely the power of the leucocytes to engulf the bacteria. The patient's temperature, on the other hand, affords a measure of the net result of many different internal processes, including the actions both of bacterial toxins and of human-cell toxins. The book is well worth reading and studying; we only wish it were a more convenient shape, possessed an index, and had its title lettered upon the back so that one could see the name when the book is on its shelf.

TUBERCULIN IN DIAGNOSIS AND TREATMENT: a Text-book of the Specific Diagnosis and Therapy of Tuberculosis for Practitioners and Students. By Dr. BANDELIER and Dr. ROEPKE. Translated from the second German edition by E. C. MORLAND, M.B., B.Sc., Lond., M.D. Berne. (London: John Bale, Sons and Danielsson, Ltd. Pp. 182 + vi., with coloured plate and charts. Price 7s. 6d. net.)

ALWAYS the chief argument of those who advocate the routine use of tuberculin in tuberculosis is the fact that well-known workers like Trudeau, Turban, and Moeller have stated that patients thus treated show years afterwards better results than others. What receives too little of their attention is the extent of precaution taken against fallacies. One finds in this work, as usual, all the *pros* and very few of the *cons*. Competence in interpreting statistics being granted (rather a large admission), what of the guarantees that the use of tuberculin was the only varying factor? Certainly the clinical material of Drs. Bandelier and Roepke is not such as to afford a test, since they expressly stipulate for selection of cases. Bad general condition, pyrexia, severe mixed infections—all these things are held to contra-indicate the use of tuberculin. And the *naïve* statement—"We have repeatedly observed a pulse-rate of 120 in the minute fall steadily to 80 in the course of six months" shows how curiously blind to the possibility of the best known of fallacies can be those who speak justly of medicine as an applied science, and have, besides, long experience of the natural remissions of consumption. This criticism, however, apart—and no sweeping condemnation of tuberculin treatment is intended—we cannot but praise this book highly. As the authors point out, its value lies in its universality. The ground indicated by its title has been covered thoroughly, with full cognisance of the multitudinous results obtained in this field by Continental workers, and of the theories upon which such work is based. This being so, some detailed mention of the authors' con-conclusions is called for. Profiting by the early errors of their master Koch, they are all for the mild, or "reactionless," method of tuberculinisation, beginning with $\frac{1}{1000}$ milligram of new tuberculin (bacillary emulsion), not going beyond 10 milligrams; by "reaction" they mean not only slight pyrexia—which should be avoided—but even mere headache and lassitude. Individual variations in susceptibility must be carefully studied. Subcutaneous administration is the only useful method. As regards diagnosis, they condemn the ophthalmo-reaction as dangerous (in ocular disease), and also, on the testimony of several observers, as untrustworthy. On the other hand, they think highly of v. Pirquet's skin reaction, and of subcutaneous injection. But here again they fail to point out that the scope of the latter means of diagnosis is much limited, since pyrexia contra-indicates it; and also that for the humiliating reason that ease of application has caused the ophthalmo-reaction to be more used than v. Pirquet's test, the former has had a more exacting trial. Dr. Morland's translation is above the usual level although (perhaps through conscientiousness) out-of-the-way words are occasionally used and German constructions retained. He states that discretion was given him to add material suitable for an English edition—*i.e.* a criticism of opsonin treatment in pulmonary tuberculosis. Although he has good warrant for doing this, he shows wisdom in refraining.

### DISEASES OF THE HEART.

Graphic Methods in Heart Disease. By John Hay, M.D., M.R.C.P. (Oxford Medical Publications. London: Henry Frowde, and Hodder and Stoughton. Pp. xviii and 184. Price 7s. 6d. net.)

The work of Dr. James Mackenzie upon the heart and the discoveries he has made by the use of simultaneous sphygmographic records from the radial pulse, the jugular vein in the neck, and possibly also from the cardiac impulse and from the liver, have become so well known that one has hitherto expected to hear of these things almost solely from him. He now has a number of disciples, however, and some of these disciples are beginning to write upon their master's subject. It is contended that whereas sphygmographic traces from the radial pulse are of little value, much clinical information may be obtained by the multiple simultaneous records we have just mentioned. This may be so, but our own impression is, that whilst such records certainly have led to valuable scientific discoveries, and may continue to lead to others equally valuable, the procedure, no matter how carefully its details may be explained in a book like the one before us, must remain too complicated for use in general practice. Moreover, the discoveries made by the methods seem to us to be much less of clinical and practical importance than they are of scientific interest, at any rate at present. We should be very sorry if there were no workers who, using either Dr. Mackenzie's own larger book, or this abstract of it—which is what Dr. Hay's work amounts to—did not continue the scientific discoveries which Dr. Mackenzie and his pupils have been making; for out of them in time to come concrete and practically useful deductions will be drawn; but much as we regret saying so, we feel sure that the methods employed must be those of the few rather than those of the generality of practitioners. In Dr. Hay's book, after preliminary chapters upon some anatomical considerations of the heart and vessels, he passes on to a detailed account of the polygraph and other instruments; describes normal sphygmograms and their interpretation, devotes a chapter to the auricular type of venous pulse, another to the various types of extra-systole—the auricular extra-systole, the ventricular extra-systole, and the nodal extra-systole; and then discusses disturbances of the functions of the heart under the headings of Disturbances of Conductivity, Disturbances of Excitability, Disturbances of Contractibility, of Stimulus Treatment, and of Tonicity. He next deals with the difficulties in interpretation of sphygmograms, difficulties which may puzzle even the most expert; and finally ends with an illustrative case and a fairly good index. The diagrams in the work are excellent, and so also is the printing, as it is in all the Oxford Medical Publications. We only regret that the publishers have not made their books all of uniform size, for at present they vary so much that they have to go upon different shelves of one's bookcase.

### TROPICAL MEDICINE.

Jamaica. Annual Report by the Superintending Medical Officer, together with the Reports on the following departments of the Medical Service of the Island, namely, the Public Hospital, the Lying-in Hospital, the Lepers' Home, and the Lunatic Asylum for the year ended March 31, 1908. (Jamaica: Government Printing Office, Kingston.)

The above gives a good idea of the type of Report published annually by many of the different British Colonies. It shows that the bulk of the work is not specially concerned with tropical diseases, but with many of the ordinary conditions and complaints prevailing at home. Tubercle, for example, is common, and seems to be on the increase, and Dr. Kerr remarks "this foul disease is very prevalent indeed, both among those who live in towns and those who live in the country." Dr. Gifford also draws attention to the prevalence of phthisis in Kingston. He states: "It seems almost incredible that no other single disease is responsible for as many deaths in Kingston as phthisis. When we consider that Jamaica possesses one of the healthiest climates in the world, and is being advertised and vaunted as a health resort, we are bound to admit that our sanitary measures require serious revision. Our general hygienic arrangements must be at fault. Food, clothing, and housing all require improvement; that the two former are to some extent factors in the incidence of phthisis may be admitted, but I feel certain that it is to the third, improper housing, especially overcrowding, that the high percentage of pulmonary diseases is mainly due; and I am further of opinion that we shall never make any headway in the reduction of the prevalence of pulmonary tuberculosis until our whole system of housing the poor is recast." Dr. Gifford might also find it useful to study the milk supply of the town, testing the cattle with tuberculin, for example, to see what percentage are infected. Of epidemic diseases chicken-pox, influenza, malaria, measles, mumps, scarlet fever, typhoid fever, and vomiting sickness prevailed. The ætiology of the latter is obscure. Two cases of blackwater fever were reported from Spanish Town. Fortunately the dreaded scourge of the West Indies—yellow fever—did not appear, and the only way to keep that disease in abeyance is to show no relaxation, but rather to stiffen the quarantine laws. One can hardly credit the statement on page 250 that a sum of £150 only has been voted on the estimates for 1908-9 for the equipment of a bacteriological laboratory at the hospital, and a sum of £100 as remuneration to the medical officer appointed to carry on this research work. One would advise the Government of Jamaica not to undertake the scheme at all rather than to do it in this inadequate manner. A properly equipped central bacteriological laboratory for the West Indies generally, to which each colony would subscribe, would perhaps be the best thing, but this would fail, of course, when urgent examinations such as for diphtheria are required. In the district reports Dr. Moseley rightly calls attention to the danger of the introduction of hydrophobia from America. A law should be passed at once to regulate the importation of dogs.

The Thirty-seventh Annual Report of the Local Government Board, 1907-1908: Supplement containing the Report of the Medical Officer. (London: Darling and Son, Ltd. 1909. Pp. 456. Price 3s. 2d.)

The volume consists of three parts, namely, first, a digest of the detailed reports and papers in the other two parts; secondly, a series of sixteen official reports upon subjects varying from the sanitary circumstances of different boroughs to the progress and diffusion of plague and cholera respectively throughout the world in 1907; and thirdly, a series of six papers upon original work done by medical men to whom grants for the purpose of research have been made by the Local Government Board.

### NURSING AND ALLIED SUBJECTS.

Handbook for Midwives and Maternity Nurses. By Comyns Berkeley, M.B. Cantab., M.R.C.P. Lond.; Obstetric Physician to the Middlesex Hospital and Senior Physician to the City Road Lying-in Hospital, Surgeon to the Chelsea Hospital for Women. New and enlarged edition. (London: Cassell and Co., Ltd. Pp. 303.)

We congratulate the author that his belief in the utility of this book has been justified by the demand for a second edition, and we agree that the addition of appendices on Cancer of the Uterus, the Revised Rules of the Central Midwives Board, and the Rules in force at the City of London Lying-in Hospital add to the value of the book. Although realising that the book as a whole is a fair *résumé* of what the midwife should know, there are many small details which we consider should not pass uncriticised. The advice as to the general hygiene of pregnancy is good, but we do not consider spirit and water a good application for the nipples, for, by hardening the surface, it must predispose to cracking. We cannot but protest against the advice that the membranes should be ruptured in accidental hæmorrhage. It may be sound treatment if good contractions are present; but otherwise it renders abortive the modern treatment by vaginal plugging, and we should expect a fatal result if the case was one of concealed hæmorrhage. We should have liked to see some more definite instructions given so that the midwife might know when the placenta has left the uterus in the third stage of labour. This is the essence of the proper management of the third stage. The author's explanations of the mechanism of normal labour are very inadequate, and in attempting to be brief he has failed to make himself clear. We cannot believe that extended legs cause impaction of the breech because the head cannot enter the brim with the legs alongside of it. Impaction of the breech occurs long before the head is anywhere near the brim. We should have thought the splinting action of the legs in preventing lateral flexion was much nearer the truth. The chapters on the infant are well done on the whole, but we notice that the midwife is advised to send for a doctor if bleeding occurs when the cord separates. We quite agree; but some instructions should be given showing how hæmorrhage may be arrested in the meantime, for the infant may bleed to death before the doctor's arrival.

Syllabus of Lectures on Home Nursing. By A. Birch. (London: The Scientific Press, Ltd. 1909. Price 6d. net.)

Home nursing is now a subject recognised by all county councils as suitable for Educational Extension lectures. The handy syllabus contained in the publication under notice is one that has been actually in use as the basis of such a course of thirteen lectures. The brief notes and headings into which the matters dealt with are arranged are eminently calculated, as the author intends, to assist both the lecturer in delivering the lecture and the student in expanding and writing up her notes. To each and all of those who attend home-nursing classes in either capacity this inexpensive pamphlet can be strongly recommended.

### EUGENICS.

Parenthood and Race Culture. By C. W. Saleeby, M.D. Pp. 300. London: Cassell and Co., Ltd. Price 7s. 6d. net.)

However deeply we may appreciate the essential soundness of a campaign against the carelessness and neglect which are too often associated with motherhood and child-nurture, we must strongly deprecate the presence of a number of offences against good taste, and of a still greater number of offences against good English, in a work which aims at the removal of social evils and the betterment of the race. We feel that Sir Francis Galton, for instance, would prefer not to be described in the dedication as "the august master of all Eugenists," and that the various authors whose books Dr. Saleeby recommends are better without his ludicrous introductory puffs. If Dr. Saleeby could have cleared his mind of pose and his pen of "journalese," the present volume would be a useful, if unoriginal, contribution to sociology. To say that his work is based upon the idea of *selection for parenthood,* which idea he very generously fathers upon Charles Darwin, seems to us ill-advised. "Every generation is epoch-making," is typical of the sententious epigrammatic phrases with which this book is so lavishly decorated. There can be no doubt about the necessity for "serious investigation of the facts of death-rate and birth-rate." It is quite possible, if not quite probable, that the unfit, from the national point of view, are increasing more rapidly than the fit, and a movement for ascertaining how far facts bear this out is to be welcomed. But we cannot help feeling that the method and manner of this work will not inspire confidence in the author amongst the thoughtful and educated sections of the public, or draw serious attention to the problem. The division into theoretical and practical eugenics is lucid and sound. The subjects discussed are well chosen and certainly important. But we must repeat that the method and the style are, in our opinion, deplorable. It is not unfair to describe the book as an "olla podrida" of other men's work and other men's ideas, selected and arranged by the active brain and decorated by the facile pen of Dr. C. W. Saleeby.

### MATERIA MEDICA AND THERAPEUTICS.

Southall's Organic Materia Medica. By John Barclay, B.Sc., F.C.S. Revised and enlarged by E. W. Mann. Seventh edition. (London: J. and A. Churchill. Price 7s. 6d. net.)

The publication not long since, by the Pharmaceutical Society, of the British Pharmaceutical Codex has to a considerable extent removed the necessity for other text-books and practical works upon materia medica, so that although, when it was first brought out in 1874 by Mr. Southall, the work before us was of great value to medical students and practitioners and pharmaceutists, we cannot help thinking that this usefulness has now been to a considerable extent superseded. Nevertheless, no work upon the subject being perfect, there is probably room for all, and the arrangement of this volume and its limitation to organic substances are sufficiently characteristic to make us think that the book will be particularly useful to teachers of pharmaceutical subjects and to pharmaceutical and medical students.

How to Cut the Drug Bill. By A. Herbert Hart, M.D. Pp. viii.+47. (London: John Bale, Sons and Danielsson. 1909. Price 2s. 6d. net.)

We do not like the style of this publication. It is possible that of the many prescriptions in it some may be very useful, but we have so little to say in favour of the pamphlet as a whole that we prefer to refrain from criticising it in detail.

### MISCELLANEOUS ITEMS.

The Interpretation of Radium. By Frederick Soddy, M.A. "The Progressive Science Series." (London: Murray. Pp. 250. With illustrations and index.)

This book contains the substance of six free popular experimental lectures delivered at the University of Glasgow in the year 1908. It is not too much to say that Mr. Soddy has produced a work which is admirable in every way. It is written in an easy, lucid style, and is free from a wealth of technical detail, with which the layman cannot expect to make himself acquainted. The first chapter deals with the phenomenon of radio activity. The eighth chapter discusses the parentage of radium, the intermediate chapters dealing with $\alpha$ $\beta$ $\gamma$ rays and the radium emanation. The sequence is logical and sound, in fact, the arrangement of the book could not be bettered, when we consider that it is essentially popular. Mr. Soddy, however, does not confine himself to mere exposition of incoherent and unco-ordinated fact. He explains how radium seems to be an exception to our established theories of the dissipation and conservation of energy, but that in reality, "though the facts of radio-activity are revolutionary," the old hypothesis can be adjusted to embrace the theory without serious distortion. Mr. Soddy is exceptionally modest over the part which he himself has played in the experimental, as well as the theoretical, side of the study of radium. The details which he gives of the delicate experiments by which the $\alpha$ atoms can be counted indicate the great difficulty in research of this kind. We are grateful, too, to Mr. Soddy for a reference to older theories without the usual contemptuous sneer.

The Frontiersman's Pocket-book. Compiled and Edited by Roger Pocock. (London: John Murray. Price 5s. net.)

In issuing this manual under the title selected, Mr. Pocock inevitably challenges comparisons with the celebrated pocket-book upon which a good deal of Lord Wolseley's military reputation was founded. There is the essential difference that this text-book for the compleat frontiersman is the work of sixty or seventy different authors, most of whom are acknowledged as experts in some particular branch of the subject. This subdivision and specialisation is called for by the great diversity of the frontiersman's spheres of activity and training; he is expected to recognise the nationality of any given warship, and even what her armament is, to make himself equally at home and efficient in the tropics or the Arctic ice, to reduce a dislocation, or pack a transport mule, or sail a boat. The medical sections for the use of those who are not within reach of a doctor are, on the whole, good. This part of the book extends to fifty-five pages, and is the work of several authors. Probably it is in places a little too ambitious, even allowing for the practical acquaintance with some of the commoner injuries and diseases which every old frontiersman picks up. Thus it is hardly worth while to attempt a description of the symptoms and treatment of dislocation of the hip within the compass of thirteen lines. There are a few more instances in which too much has been attempted; but, on the other hand, there are very few paragraphs indeed to which any exception can be taken on the score of inaccuracy. The section on artificial respiration for the apparently drowned follows the old Silvester routine; this might well be displaced in future editions by an account of the Schäfer method. In the treatment of malaria no mention is made of an initial purge as an important aid to quinine medication. The paragraph on veld sores is unsatisfactory; of the two remedies suggested the second is by far the better, but there are refractory cases which require antiseptic baths and fomentations. It is perhaps scarcely the function of a medical journal to criticise frontiercraft as laid down by experts. But it may not be impertinent to remark on the very high standard of excellence of this pocket-book, which contains within reasonable compass a quantity of information astonishing in variety and value. It may be supposed that the author who recommends boiling to rid clothing of lice has had occasion to try it; but certainly on the high veld it is of no use, perhaps because water boils at lower temperatures in high altitudes. Another practical point, possibly considered too elementary to need mention, is to place a camp kitchen or fire to leeward of all tents, especially when the grass is dry. But we repeat that, page in page out, "The Frontiersman's Pocket-book" is thoroughly admirable.

The Climate of Strathpeffer. By H. W. Kaye, M.D. Oxon. Pp. 64. (London: Swan, Sonnenschein and Co., Ltd.)

After dealing as a whole with climate and its factors, meteorological and general, the author treats of the climate of Strathpeffer in particular. He supports, from meteorological observations extending over thirty-five years, the contention that the general characteristics of the climate of Strathpeffer are, mildness and equability, without extremes of heat or cold; relative dryness; with absence of strong winds; great purity of air, and long summer and short winter days. He recommends the place as a health resort to those suffering from chronic metabolic disorders, anæmia, chronic rheumatism, chronic lung troubles (especially emphysema), and many types of surgical tuberculosis. The best weather is experienced between April and the end of October.

Wastage of Child Life. By J. Johnston, M.D. Edin. The Fabian Socialist Series, No. 7. Pp. 96. (London: A. C. Fifield. Price 6d. paper; cloth 1s.)

Written from a frankly socialistic standpoint, this little volume deals briefly with the many problems which affect the birth, upbringing, education, and surroundings of the Lancashire slum child. Few, however, will dispute the author's opinion that many of the existent evils are the result of parental ignorance, intemperance, and improvidence. Few will disagree with him in condemning the half-time system, with its consequent stultification of the child-mind at the most receptive and impressionable period of its existence. It is only when he proposes the substitution of State for parental responsibility that he is likely to meet with serious opposition. The volume can be recommended to all who wish for a concise and readable account from a definite point of view of a great social evil.

Golden Rules of Anæsthesia. By R. J. Probyn-Williams, M.D. (Bristol: J. Wright and Company. Third edition. 1909. Price 1s.)

Much as there is to be said against the distillation of medical lore into such very concentrated essences as "Golden Rules," it is to be frankly conceded that in Dr. Probyn-Williams' hands the process is less objectionable than could be thought possible. It is true enough that only practice and intelligent study of actual administrations can make a man into an anæsthetist; but the last-year student who remembers and obeys the very explicit rules of anæsthesia set down in this pamphlet will be, at least potentially, an anæsthetist of much more than average capacity. That those for whom it is designed appreciate this is sufficiently attested by the appearance of this, the third edition.

